# Cost-effectiveness analysis of human papillomavirus DNA testing and Pap smear for cervical cancer screening in a publicly financed health-care system

**DOI:** 10.1038/sj.bjc.6605974

**Published:** 2010-11-23

**Authors:** I H-I Chow, C-H Tang, S-L You, C-H Liao, T-Y Chu, C-J Chen, C-A Chen, R-F Pwu

**Affiliations:** 1 School of Health Care Administration, Taipei Medical University, Taipei, Taiwan; 2 Gynecology Research Center, Taipei Medical University Hospital, Taipei, Taiwan; 3 Genomics Research Center, Academia Sinica, Taipei, Taiwan; 4 School of Public Health, National Defense Medical Center, Taipei, Taiwan; 5 Institute of Health Policy and Management, National Taiwan University, Taipei, Taiwan; 6 Center for Drug Evaluation, Taipei, Taiwan; 7 Department of Obstetrics and Gynecology, Buddhist Tzu Chi General Hospital, Tzu Chi University, Hualien, Taiwan; 8 Center of Cervical Cancer Prevention, Buddhist Tzu Chi General Hospital, Tzu Chi University, Hualien, Taiwan; 9 Graduate Institute of Epidemiology, College of Public Health, National Taiwan University, Taipei, Taiwan; 10 Department of Obstetrics and Gynecology, College of Medicine, National Taiwan University, Taipei, Taiwan

**Keywords:** HPV DNA testing, Pap smear, cost-effectiveness analysis, cervical cancer, screening

## Abstract

**Objective:**

To evaluate the long-term cost-effectiveness of different strategies for human papillomavirus (HPV) DNA testing combined with Pap smear for cervical cancer screening in Taiwan.

**Methods:**

This study adopts a perspective of Department of Health in cost-effectiveness analysis to compare a no-screening strategy with nine different screening strategies. These strategies comprise three screening tools (Pap smear alone, HPV DNA testing followed by Pap smear triage, and HPV DNA testing combined with Pap smear), and three screening intervals (annually, every 3 years, and every 5 years). Outcomes are life expectancy, quality-adjusted life years (QALYs), lifetime costs, and incremental cost-effectiveness ratios (ICERs). Probabilistic sensitivity analyses (PSAs) were conducted to assess parameter uncertainty.

**Results:**

When three times gross domestic product per capita is used as the decision threshold, all nine screening strategies were cost-effective compared with the no-screening strategy. Compared with the current screening strategy (an annual Pap smear), HPV DNA testing followed by Pap smear triage every 5 years and every 3 years were cost-effective. Results of PSA also indicated that a HPV DNA testing followed by Pap smear triage every 5 or every 3 years achieved the highest expected net benefits.

**Conclusions:**

Possible economic advantages are associated with extending the cervical cancer screening interval from one Pap smear annually to HPV DNA testing followed by Pap smear triage every 5 years with an ICER $1 247 000 per QALY gained, especially in a country with a publicly financed health-care system.

Cervical cancer is one of the most common cancers in Taiwan, currently ranked as the fifth leading cancer causing death for females (Chen *et al*, 2009). Although the incidence and mortality associated with cervical cancer have declined steadily since the National Health Insurance (NHI) program was instituted in 1995, the incidence of cervical cancer in Taiwan is still one of the highest in Asia and is a huge burden to the health-care system.

All females in Taiwan aged ⩾30 years are now provided with a free Pap smear annually by the NHI system (Koong *et al*, 2006). The annual Pap smear screening rate for females aged 30–69 years increased to 27.4% in 2007 from 14.3% in 1996. The 3-year coverage rate reached 52.4% during 2005–2007; 5-year coverage was roughly 60% during 2003–2007 (Bureau of Health Promotion, 2007). In France, 60% of the female population had a regular Pap smear within the last 3 years (Schaffer *et al*, 2000), 80% of Dutch females and 85% of English females had a Pap smear within the last 5 years (Patnick, 2000; Van Ballegooijen and Hermens, 2000). Notably, the screening program in Taiwan has not yet achieved satisfactory coverage. The health authorities in Taiwan currently face the challenge of implementing cervical cancer screening programs with increased effectiveness.

A population-based randomised trial conducted in Sweden indicated that primary the human papillomavirus (HPV) DNA testing with Pap smear triage and repeat HPV DNA testing for those who test positive in HPV DNA testing with negative Pap smear results were the most effective cervical cancer screening strategy (Naucler *et al*, 2009). Recent findings from a cohort study on long-term outcomes of HPV infection in Taiwan demonstrated that, for a female who had a normal Pap smear result, the 6-year cumulative risk of developing high-grade squamous intraepithelial lesions (HSILs) was 10.0% when the HPV DNA testing result was positive, whereas the risk for those with negative HPV DNA testing was only 0.34%. These data support the efficacy of HPV DNA testing for cervical cancer screening program with intervals of at least 5 years (Huang *et al*, 2008). Another European study of six countries also suggested that screening intervals could safely be lengthened to 6 years among females with a negative HPV DNA testing result (Dillner *et al*, 2008).

In two systematic review studies, Holmes *et al* (2005) and Muhlberger *et al* (2008) summarised existing evidence for the cost-effectiveness of incorporating the HPV DNA testing into current cervical cancer screening programs. Their analytical results showed the HPV DNA testing with a Pap smear as a screening strategy for females aged ⩾30 years was cost-effective. With an incremental cost-effectiveness ratio (ICER) threshold of €50 000 per quality-adjusted life year (QALY), Muhlberger *et al* (2008) concluded that, in comparison with annual Pap smear, or Pap smear every 2, 3, or 5 years, the probabilities of HPV DNA testing strategies being cost-effective were 83, 25, 55, and 92%, respectively. These analytical findings illustrate that when evaluating the cost-effectiveness of the HPV DNA testing, the screening interval should be considered.

Several studies utilised computer-based models to estimate the cost-effectiveness of adding the HPV DNA testing to current cervical cancer screening programs in Sweden, Canada, the United States, the United Kingdom, the Netherlands, France, Italy, and Germany. Adding the HPV DNA testing can reduce cancer incidence and is less costly than Pap smear alone (Mandelblatt *et al*, 2002b; Mittendorf *et al*, 2003; Goldie *et al*, 2004; Kim *et al*, 2005; Bistoletti *et al*, 2008; Chuck, 2009).

In Taiwan, which has a poor Pap smear participation rate, policy makers face the problems of whether to combine the HPV DNA testing with Pap smear for cervical cancer screening and determining the optimal screening interval. This study evaluates the long-term cost-effectiveness of different strategies for HPV DNA testing combined with Pap smear for cervical cancer screening in Taiwan for healthy females aged 30 years.

## Materials and methods

### Cost-effectiveness analysis and decision analytic model

This cost-effectiveness analysis (CEA) adopts the perspective of Department of Health. A decision analytic model was developed using TreeAge Pro Suite 2009 software (TreeAge Software, Inc., Williamstown, MA, USA) for evaluating the long-term costs and effectiveness of various cervical cancer screening strategies. This study used clinical outcome measures, including cervical cancer incidence, mortality, life expectancy, and QALYs. All costs, life expectancy, and QALYs were discounted to their present values at an annual rate of 3%. The ICERs were calculated as the difference in cost divided by the difference in QALYs for each strategy compared with the no-screening strategy, with the strategy of an annual Pap smear, or with the next-best strategy. Currently, no consensus exists for a specific cost-effectiveness threshold in Taiwan. However, this study considered strategies with ICER values less than three times per capita gross domestic product (GDP) as cost-effective (Eichler *et al*, 2004). In 2008, Taiwan's per capita GDP was NT$538 790 (Directorate-General of Budget, Accounting and Statistics, 2009a). Therefore, the cost-effectiveness threshold assumed in this model was NT$1 620 000/QALY.

### Natural history model

The natural history model for cervical cancer was adopted from literature (Debicki *et al*, 2008; Rogoza *et al*, 2008; Suárez *et al*, 2008). The health states of the decision analytic model for the natural history of cervical cancer among females have the following seven categories: normal (healthy); HPV infection; grades of cervical intraepithelial neoplasia (CIN) (CIN I, CIN II or III); persistent CIN II or III; stage of cervical cancer; death from other causes; and, death from cervical cancer. This model simulates a cohort of 30-year-old healthy females; the hypothetical cohort moves between different health states according to prespecified probabilities in each Markov cycle (1 year). The analytical time horizon was lifetime (85 years old).

The model starts with a cohort of 30-year-old healthy females (normal state). During simulation, individuals may remain in the normal state or progress to the HPV infection state in the next year (or cycle). Individuals with HPV infection may remain in the HPV infection state, regress to the normal state, or progress towards the CIN I state. The CIN I patients may remain in the CIN I state, regress to the normal state, or progress towards the CIN II or III state. The CIN II or III patients may remain in the CIN II or III state, regress to the normal state, or progress towards the persistent CIN II or III state. From there, patients may remain in the persistent CIN II or III state, regress to the normal state, or progress towards the cervical cancer state. When individuals reach the cervical cancer state, we assume no regression is possible; that is, they can only progress to a cured cancer state when alive at 5 years after diagnosis or to the cervical cancer death state. In this model, a female in any state can die because of other causes.

### Screening strategies

This study was designed to compare a no-screening strategy with nine different screening strategies, which varied using three different screening algorithms – Pap smear alone, HPV DNA testing followed by Pap smear triage, and HPV DNA testing combined with Pap smear, with three screening intervals – annually, every 3 years, and every 5 years. We assume the ceiling age for screening is 69 years.

Table 1 lists the primary screening and triage tool, screening ages, screening interval, and coverage for each strategy. We assume a 30% coverage rate for annual screening, based on the reported 28.4% of females aged 30–69 years who had a Pap smear in 2007. Similarly, 3-year (60%) and 5-year coverage rates (75%) were assumed as the reported percentages of 52.5% and 64.9% of those who underwent at least one Pap smear during a 3- or 5-year period, respectively.

**Figure 1:**
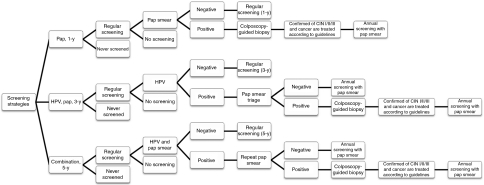
Screening decision tree showing different screening tests at three screening intervals and follow-up management.

When a female is invited to undergo cervical cancer screening, she is stratified into the never-screened or regularly screened categories (Figure 1). According to research by Chen *et al* (2009), after implementing the screening program in 1995, roughly 75% eligible females in Taiwan participated in the program at least once by 2007. We infer that the percentage of those never screened was 25%. Considering the uncertainty, 12% and 30% as low and high estimate values were changed during sensitivity analysis.

In the regular screening strata, a female may or may not be willing to participate in an appropriate year. If a female chooses to undergo screening, the following three combinations of screening tools were evaluated: (1) Pap smear as the primary screening test followed by colposcopy-guided biopsy for positive Pap smear results (i.e., Pap); (2) HPV DNA testing as the primary screening test, using a Pap smear as a triage strategy for those who test positive for high-risk HPV types in HPV DNA testing (i.e., HPV-Pap); and, (3) HPV DNA testing in combination with Pap smear as the primary screening test, using a Pap smear as a follow-up screening strategy for those who have positive Pap smear results or test positive for high-risk HPV types (i.e., combination).

Experts and existing guidelines were consulted before making the following assumptions for management of females with different screening results: (1) women with negative primary screening results are asked to undergo regular screening; (2) when a woman had a positive final screening result, she is urged to undergo a colposcopy-guided biopsy. All women with a confirmed histology of CIN I, CIN II or III and cancer are assumed to be treated with the therapy recommended by the Taiwan Cooperative Oncology Group (Taiwan Cooperative Oncology Group, 2007). These patients are assumed treated immediately after disease confirmation. When treatment was completed, annual Pap smear screening followed; (3) in the HPV-Pap and combination strategies, those negative after follow-up screenings are suggested to undergo annual Pap smear screening for the rest of their lifetime.

We further assume that compliance with follow-up and treatment is 100% in the model.

### Model parameters

Table 2 summarises the probabilities, costs, and data sources used in the base-case analysis.

#### Parameters of the natural history model

Parameters of HPV infection rates and disease transitions were obtained from the study by Debicki *et al* (2008). They estimated disease parameters by reviewing published reports of Taiwanese screening coverage and cancer incidence, and further calibrated disease parameters to fit cervical endpoints. The succinct model, which adopted fewer complex processes and a short cycle length of 1 year, was used in this study.

#### Survival rate and death probability

The annual probabilities of dying from cervical cancer for patients were obtained from survival rates published by the Taiwan Cancer Registry (2007). The 1-, 2-, 3-, 4-, and 5-year survival for patients with invasive cervical cancer (all stages) in Taiwan were 90.4, 83.2, 79.4, 76.7, and 74.8%, respectively.

Age-specific other-cause mortality was obtained from the 2007 Life Table for Taiwan's female population (Department of Statistics, 2008). For example, the probability of dying, which increases with age, was 0.00055 for those aged 30 and 1 for those aged 85.

#### Screening test characteristics

Test sensitivity was defined as a positive screening result for patients with CIN I or worse in this model. Test specificity was defined as a negative result for females in the normal state. Screening test characteristics used were obtained from and Mayrand *et al* (2007). Mayrand *et al* conducted a randomised trial comparing the sensitivity and specificity of the HPV DNA testing and Pap smear for females aged 30–69 years in Canada (*n*=10 154). This study used Pap smear sensitivity for CIN II or III of 55.4% (95% CI=33.6–77.2%), and a specificity of 96.8% (95% CI=96.3–97.3%). Arbyn *et al* conducted meta-analyses of clinical applications of the HPV DNA testing. Their analytical results indicated that sensitivity of the HPV DNA testing (89.5% 85.1–93.1%) or the combination of the HPV DNA testing with Pap smear (99.2% 97.4–100.0%) are higher than Pap smear alone in other studies for detecting CIN II cases or worse. The specificity was 87.5% (95% CI = 85.0–89.9%) for the HPV DNA testing and 87.3% (95% CI=87.3–90.4%) for the combination test.

#### Utility weight data

The health utility data for the base case and ranges were adopted from a CEA evaluating HPV vaccination programs in Taiwan (Suárez *et al*, 2008), which was compiled using health utility data collected in the USA (Insinga *et al*, 2007).

#### Costs data

From a perspective of Department of Health, only costs associated with direct medical costs were utilised. Furthermore, this study included only the direct medical costs associated with use of health-care services covered by the NHI program.

The category for costs associated with cervical cancer consists of costs of screening tests, costs of diagnosis, costs of treatment for CIN I, CIN II or III, and cancer treatment costs.

#### Screening costs

Pap smear screening as a procedure for cervical cancer prevention includes: (1) Pap smear sampling, (2) pelvic examination, and (3) Pap smear cytological examination. Health-care providers are reimbursed NT$430 by the NHI system for these services (Bureau of National Health Insurance, 2009). Additionally, the average price of the HPV DNA testing is assumed to be NT$600.

#### Diagnostic costs

For the HPV-Pap and combination strategies, patients who had a positive primary screening test underwent another Pap smear test. The procedures for a repeated Pap smear are (1) vaginal irrigation, (2) pelvic examination, (3) obtaining Pap smear sample, and (4) Pap smear cytological examination. Health-care providers are reimbursed NT$440 by the NHI system for these services (Bureau of National Health Insurance, 2009). Those who had a positive primary screening test with the Pap strategy, or had a positive final screening result with the HPV-Pap or combination strategy, or have been with cervical cancer or precancerous lesion, are referred to take a colposcopy-guided biopsy. Diagnostic cost of the colposcopy-guided biopsy was NT$2183 (Debicki *et al*, 2008).

#### Treatment costs

Direct medical costs for precancerous lesions and cervical cancer treatments in Taiwan were obtained from the study by Tang *et al* (2010). All costs are reported in New Taiwan Dollars (in this study, US$1.00=NT$33.032).

### Sensitivity analysis

Considering uncertainty is crucial in a decision analytical study. To better understand the impact on the results for different levels of participation, along with the purpose to present the validity of the model results, a scenario sensitivity analysis was performed by approximating the highest coverage rate at 100% for all screening intervals. Probabilistic sensitivity analyses (PSAs) were conducted to assess parameter uncertainty. The ranges of parameters were mostly from published studies (Table 2). A 50% increase or decrease in the base-case value for costs data were changed during sensitivity analysis.

Probability distributions were defined for the following six sets of model parameters: (1) screening rate; (2) disease transition probabilities in the natural history model; (3) survival rate for cervical cancer; (4) test characteristics; (5) utility; and, (6) costs. A beta distribution was fitted for screening rate, the natural history model, survival rate for cervical cancer, test characteristics, and utility, those parameters were between 0 and 1. A gamma distribution can be used to represent uncertainty in cost data, which was constrained to be non-negative and characterised in the interval of 0 to positive infinity (Briggs *et al*, 2007).

The PSA results in this model were analysed using cost-effectiveness acceptability frontier (CEAF) constructed from cost-effectiveness acceptability curves (CEACs) for the different strategies. The CEACs were calculated from net monetary benefit over a range of willingness-to-pay (WTP) thresholds for each strategy, and produced by plotting the probability of being cost-effective for a strategy under different WTP thresholds (Barton *et al*, 2008). To further determine the uncertainty associated with *a priori* intervention over all WTP thresholds for each strategy (Fenwick *et al*, 2001), this study produced a CEAF by identifying the probability of the optimal strategy in each of 1000 iterations based on the strategy with the highest expected net benefits at different WTP thresholds (Barton *et al*, 2008).

## Results

### Base-case analysis

Table 3 presents the lifetime effectiveness and costs for a hypothetical cohort of 30-year-old females for all nine screening strategies. Predicted effectiveness outcomes were lifetime incidence and mortality for cervical cancer, total life expectancy, and total QALYs; additionally, total lifetime costs, and ICERs calculated by comparing different bases (i.e., the no-screening strategy, Pap annual strategy, and next-best strategy) are presented.

#### Reduction in lifetime risk

Compared with the no-screening strategy, all nine screening strategies were more effective (Table 3). The estimated reduction in lifetime incidence and related mortality for cervical cancer when screened at 30 years of age varies at 35.8–71.1%, depending on screening tests, screening intervals, and screening strategies. The HPV-Pap and combination strategies at annual, 3-, or 5-year intervals prevented more cases than the Pap strategy at annual, 3-, or 5-year screening intervals. The model estimated cervical cancer cases at 87.6–118.2 per 100 000 women and a related mortality of 25.6–34.8 per 100 000 patients with the Pap strategy; 54.6–70.0 cases and 16.3–20.7 deaths for the HPV-Pap strategy; and 53.2–65.2 cases and 15.9–19.3 deaths with the combination strategy (Table 3).

#### Cost-effectiveness analysis

**Figure 2:**
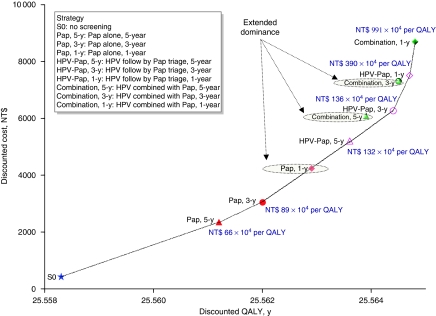
Base-case efficiency frontier depicting costs and QALYs for cervical cancer screening strategies. The 1-year (30%), 3-year (60%), and 5-year coverage rates (75%) were assumed those who underwent at least one Pap smear during a 1-, 3-, or 5-year period.

Figure 2 presents the cost-effectiveness results that indicate that all nine screening strategies generated greater effectiveness (i.e., total QALYs) and total costs than the no-screening strategy from the perspective of Department of Health. Using three times Taiwan's per capita GDP as the cost-effectiveness threshold, all these strategies were cost-effective. However, when each strategy compared with the next most-effective non-dominated strategy, an annual Pap, combination every 5 years and every 3 years were extended-dominated. Pap every 5 years and every 3 years, HPV-Pap every 5 years and every 3 years had ICERs of NT$660 000, NT$889 000, NT$1 323 000, NT$1 358 000 per QALY gained, respectively. These strategies were cost-effective at three times per capita GDP threshold.

If the current screening strategy, an annual Pap, is used for comparison, HPV-Pap every 3 years and every 5 years cost NT$1 303 000 and NT$1 247 000 per QALY gained, respectively. These strategies are <NT$1 620 000/QALY. However, extending screening intervals for Pap annually to every 3 or 5 years was less costly but also less effective. Changing Pap annually to every 3 or 5 years save NT$1197 to NT$1890 while loss 0.33–0.62 of discounted quality-adjusted life days per woman (see Table 3 and Figure 2).

### Sensitivity analysis

**Figure 3:**
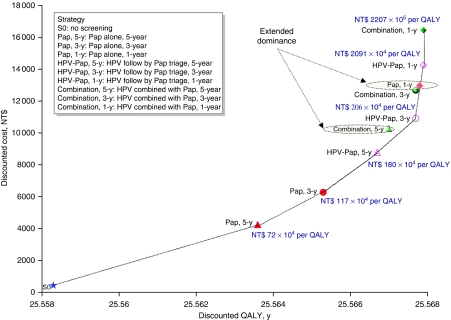
Sensitivity analysis efficiency frontier depicting costs and QALYs for cervical cancer screening strategy. Screening coverage rate was assumed 100% for all screening intervals.

The coverage rate changes into 100% for all screening intervals in the scenario sensitivity analysis. Results show that screening strategies on the sensitivity analysis efficiency frontier in this analysis are Pap every 5 years and 3 years, HPV-Pap every 5 years, every 3 years and annually, and combination annually (Figure 3). The ranking, and the magnitude of the discounted QALYs and costs, of the alternatives are as expected (i.e., programs with the annual screening are the most efficacious and the most costly), suggesting the validity of this model.

**Figure 4:**
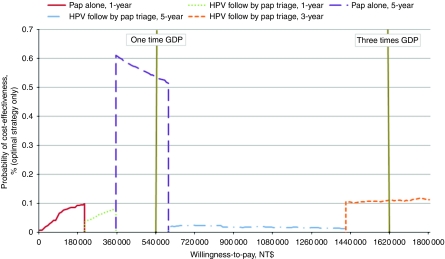
Cost-effectiveness acceptability frontier curves.

The PSA results are presented in a CEAF. The CEAF was plotted by presenting the optimal strategy under different WTP thresholds (Figure 4). When three times GDP per capita is used as the decision threshold, the annual Pap strategy (<NT$207 000), the annual HPV-Pap strategy (NT$207 000–351 000), the Pap strategy every 5 years (NT$351 000–594 000), the HPV-Pap strategy every 5 years (NT$594 000–1 413 000), and the HPV-Pap strategy every 3 years (⩾NT$1 413 000) achieved the highest expected net benefits.

## Discussion

Screening has proven cost-effective in preventing cervical cancer according to many studies conducted in different countries (Mandelblatt *et al*, 2002a, 2002b; Mittendorf *et al*, 2003; Goldie *et al*, 2004, 2005; Kim *et al*, 2004, 2005; Holmes *et al*, 2005; Siebert *et al*, 2006; Anderson *et al*, 2008; Andres-Gamboa *et al*, 2008; Bistoletti *et al*, 2008; Muhlberger *et al*, 2008; Chuck, 2009; Vijayaraghavan *et al*, 2009); the model results in this study demonstrate the cost-effectiveness of screening compared with that of a no-screening strategy, regardless of which screening tool or interval is used. Compared with the annual Pap strategy, the HPV-Pap strategy at 3- or 5-year intervals are cost-effective. Annual HPV-Pap strategy, combination strategy annually or at 3- or 5-year intervals generated the greatest effectiveness, while cost a substantial amount of total costs; thus, the annual HPV-Pap and the combination strategies are not cost-effective. For a nation with a publicly financed health-care system, such as Taiwan, this study shows that extending cervical cancer screening interval from Pap annually to HPV-Pap strategy every 3 or 5 years costs NT$1 303 000 or NT$1 247 000 per QALY gained, respectively.

Compared with the no-screening strategy, Pap annually and Pap every 3 and 5 years reduced the incidence of cervical cancer in a lifetime by 52.4, 44.1, and 35.8% under the coverage assumptions in this model. Arbyn *et al* (2006) and Mayrand *et al* (2007) indicated that the HPV DNA testing has greater sensitivity and is more accurate than Pap smear for detecting HSILs. As expected, analytical results demonstrate that the HPV-Pap or combination strategy annually or at 3- or 5-year screening intervals prevented more cases than the Pap strategy annually or at 3- or 5-year screening intervals. The reductions in lifetime incidence of cervical cancer with the HPV-Pap strategy annually or at 3- or 5-year screening intervals were 70.3, 67.4, and 62.0% the values for the combination strategy were 71.1% (annually), 69.0% (every 3 years), and 64.6% (every 5 years). Two studies undertaken in the United States reported a decline in lifetime incidence of cervical cancer of 70–87% (Mandelblatt *et al*, 2002b) and 81–90% (Goldie *et al*, 2004) for Pap smear alone with different screening intervals. The HPV DNA testing with a Pap smear in a cervical cancer screening program decreased risk by 78–93% (Mandelblatt *et al*, 2002b) and 90–93% (Goldie *et al*, 2004). This study identified an increasing trend of a reduction in the incidence of cervical cancer that is consistent with findings obtained with other models comparing the HPV DNA testing with Pap smear alone.

Several studies demonstrated that using the HPV DNA testing as primary screening or combined with Pap smear with a screening interval of every 2, 3, or 5 years was more cost-effective than Pap smear alone (Mandelblatt *et al*, 2002b; Mittendorf *et al*, 2003; Goldie *et al*, 2004; Kim *et al*, 2005; Bistoletti *et al*, 2008; Chuck, 2009). Analytical results in this study demonstrate that the HPV-Pap strategy at 3- or 5-year intervals was cost-effective; this finding is generally consistent with those studies. However, the results of this model demonstrate that the combination strategy, regardless of screening intervals, were not cost-effective compared with current cervical cancer screening program (Pap annually). Three important factors were considered in this study. First, the combination strategy annually and at 3- and 5-year intervals were more effective (i.e., total QALYs) than Pap annually. The combination annually strategy saves 0.69 days of discounted QALY per woman compared with Pap annually. Whereas total costs of the combination strategy is more than 1.4-fold (combination every 5 years) to 2.0-fold (combination annually) that of the annual Pap strategy (NT$4242) (Table 3). Thus, extending the cervical cancer screening interval from Pap annually to HPV-Pap strategy every 3 or 5 years can be considered.

Second, differences in cost structures may account the difference between cost-effectiveness results in this study and those obtained by other studies using the HPV DNA testing as primary screening or combined with Pap smear for cervical cancer screening. The unit cost of the HPV DNA testing is 40% more than that of a Pap smear in Taiwan. Conversely, the unit cost of the HPV DNA testing is roughly identical to that of Pap smear in many European countries and in the United States (Mandelblatt *et al*, 2002b; Kim *et al*, 2004, 2005; Bistoletti *et al*, 2008). In comparison, the screening cost of the HPV-Pap and combination strategies are higher in Taiwan than in other countries.

Third, using the HPV DNA testing increased disease cases by detecting more precancerous lesions than Pap smear alone (Naucler *et al*, 2009). The costs of treatment with the HPV-Pap or combination strategy may be lower than Pap smear alone theoretically. In fact, the costs of treatments for precancerous lesions and cervical cancer in Taiwan are lower than those in many countries (Goldie *et al*, 2004; Kim *et al*, 2004, 2005; Tang *et al*, 2010). For instance, treatment cost was roughly NT$6956 for CIN II or III patients. These figures are more than three-fold in the UK (US$678, about NT$22 463) and seven-fold in the United States (US$2 833, about NT$93 863). Moreover, overall invasive cervical cancer treatment costs are 1.6-fold higher in the Netherlands (US$17 603, about NT$583 223) to 2.8-fold higher in the UK (US$31 494, about NT$1 043 443) than in Taiwan (NT$375 322). The difference in treatment cost between the HPV-Pap or combination strategy and Pap smear alone is less in Taiwan than that in other countries. Consequently, using the HPV DNA testing has the potential to improve health effectiveness at a reasonable cost compared with the Pap smear in European countries and in the United States is not surprising. Early detection of precancerous lesions may not reduce treatment costs largely; thus, it may have difficulty being cost-effective when treatment costs are generally lower in Taiwan than in other countries.

Analytical results demonstrated that extending screening intervals from Pap annually to every 3 or 5 years would save costs but be less effective; this finding is generally consistent with those in studies by Anderson *et al* (2008) and Siebert *et al* (2006). The population of women aged ⩾30 years, that is, those targeted for cervical cancer screening, was estimated at approximately 7 million in Taiwan in 2008 (Directorate-General of Budget, Accounting and Statistics, 2009b). However, the budget for the screening program (free annual Pap smear-approximately NT$430 for one Pap smear) from the Bureau of Health Promotion, Department of Health, was only NT$860 million annually (Liberty Times, 2009). Although this figure seems quite small, it hardly meets the expenses under low participation. As a result of the limited resources available for cervical cancer screening programs, implementing an annual screening program is barely affordable. The cost-effectiveness results obtained by this study suggest that HPV-Pap every 3 or 5 years represent an optimal use of resources, considering improve participation rate and extending screening intervals.

Some studies have explored factors associated with low participation for the cervical cancer screening program in Taiwan (Lee *et al*, 1997; Lin *et al*, 2003, 2007). These factors may be summarised as Taiwanese women having a more lax attitude toward cervical cancer screening programs, perhaps because of societal values or those in traditional culture. This is also at play in the difficulty policy-makers have faced during the last decade in determining how to improve coverage for Pap smear screening. One alternative to screening may be self-sampling, which may make cervical cancer screening more accessible to women not participating in the current screening program or those rarely contacting health-care centres, offering an important method for increasing cervical cancer screening coverage (Sellors *et al*, 2000; Wright *et al*, 2000; Agorastos *et al*, 2005; De Alba *et al*, 2008). Future studies should evaluate the cost-effectiveness of self-sampling for HPV DNA testing.

This study has several limitations. First, women treated for CIN I, CIN II or III may have a different (i.e., higher or lower) probability of recurrent cervical lesions than those who have negative test results. However, we did not have good evidence on this difference. Thus, we assumed that women treated for CIN I, CIN II or III were cured and returned to a normal state and acquired a new HPV infection at similar rates to those without previous abnormal results. The model assumed that women must have been infected with HPV to develop further lesions or diseases. This study did not consider disease progression without previous HPV infection.

Second, while the natural history model was based on Taiwan calibrated data sources from studies, most transition probabilities were obtained from literature with no age stratification for all Pap, HPV-Pap, and combination strategies. The model also used the same cervical cancer survival rate for all ages, which is not the case in reality. This may add to model uncertainties and parameter uncertainties in analytical results; however, sensitivity analyses demonstrate the robustness of study results.

Third, this study did not consider women age <30 years who have already infected with high-risk HPV. According to a cohort study in Taiwan, the prevalence of HPV infection was 9.09% in women age <30 years (Huang *et al*, 2008). For women with HPV infection, they are recommended to undergo a Pap smear as a triage screening. If necessary, further tests or treatments will be required. Thus, the total costs estimates in this study may have been underestimated; however, the magnitude is not likely to be high.

Finally, this study also assumed that all women with negative primary screening results were asked to undergo regular screening until 69 years of age. Although the recent scientific evidence suggests that women aged 50 or above with negative HPV test results will have a lower risk to develop cervical cancer, it is the general consensus in Taiwan that aged women should also be covered in screening programs. It will be of value, however, to explore the cost-effectiveness of polices that considered the strategies for different age groups.

In conclusion, this study demonstrates that, compared with a no-screening or an annual Pap strategy, using the HPV DNA testing in the cervical screening program in Taiwan is cost-effective. This study also identified as cost-effective for extending the cervical cancer screening interval from Pap annually to HPV-Pap strategy every 3 or 5 years had ICERs of NT$1 303 000 or NT$1 247 000 per QALY gained, respectively. Considering the potential economic advantages, HPV-Pap strategy every 5 years would seem to be more attractive, especially in Taiwan, a country with a publicly financed health-care system.

## Figures and Tables

**Table 1:** Cervical cancer primary screening and follow-up tool, screening ages, screening interval, and coverage for each strategy

**Screening strategy** a	**Primary screening**	**Follow-up screening** b	**Screening ages, year**	**Screening interval, year**	**Coverage, %** c
Pap, 1-y	Pap smear	—	30–69	1 year	30.0 (27.4–50.0)
Pap, 3-y	Pap smear	—		3 year	60.0 (52.4–65.0)
Pap, 5-y	Pap smear	—		5 year	75.0 (70.0–75.0)
HPV-Pap, 1-y	HPV DNA testing	Pap smear		1 year	30.0 (27.4–50.0)
HPV-Pap, 3-y	HPV DNA testing	Pap smear		3 year	60.0 (52.4–65.0)
HPV-Pap, 5-y	HPV DNA testing	Pap smear		5 year	75.0 (70.0–75.0)
Combination, 1-y	Combine HPV DNA testing with Pap smear	Repeat Pap smear		1 year	30.0 (27.4–50.0)
Combination, 3-y	Combine HPV DNA testing with Pap smear	Repeat Pap smear		3 year	60.0 (52.4–65.0)
Combination, 5-y	Combine HPV DNA testing with Pap smear	Repeat Pap smear		5 year	75.0 (70.0–75.0)

Abbreviation: HPV=human papillomavirus.

a ‘Pap’ refers to Pap smear as primary screening test follows by colposcopy-guided biopsy for positive Pap smear results. ‘HPV-Pap’ refers to HPV DNA testing as primary screening test, using Pap smear as a triage strategy for those who test positive for high-risk HPV types in HPV DNA testing results. ‘Combination’ refers to HPV DNA testing in combination with Pap smear as primary screening test, using Pap smear as a follow-up screening strategy for those who have Pap smear results or test positive for high-risk HPV types. ‘1-y’, ‘3-y’, and ‘5-y’ refer to a 1-year, 3-year, and 5-year screening intervals for women 30–69 years of age, respectively.

b Follow-up of positive primary screening results.

c 1-year, 3-year, and 5-year coverage rates were assumed as the reported percentages of those who underwent at least one Pap smear during 2007, 2005–2007, or 2003–2007 period.

**Table 2:** Model parameters of the decision analytic model for cervical cancer

**Model parameter**	**Base case**	**Range**	**Reference**
*Natural history*			
Normal to HPV	0.06	0–0.17	Debicki *et al* (2008);
HPV to CIN I	0.049	0.028–0.07	Rogoza *et al* (2008);
CIN I to II/III	0.091	0.08–0.30	Suárez *et al* (2008)
CIN II/III to persistent CIN II/III	0.114	0–0.114	
Persistent CIN II/III to cancer	0.05	0–0.10	
HPV regression	0.516	0.435–0.87	
CIN I regression	0.449	0.31–0.54	
CIN II/III regression	0.227	0.04–0.227	
			
*5-year survival rate from cervical cancer,* %			
1-year	90.4	85.4–95.4	Taiwan Cancer Registry (2007)
2-year	83.2	78.2–88.2	
3-year	79.4	74.4–84.4	
4-year	76.7	71.7–81.7	
5-year	74.8	69.8–79.8	
			
*Test characteristics,* %^a^			
Sensitivity of Pap smear	55.4	33.6–77.2	Mayrand *et al* (2007)
Sensitivity of HPV DNA testing	89.5	85.1–93.1	Arbyn *et al* (2006)
Sensitivity of Pap smear and HPV DNA testing	99.2	97.4–100	Arbyn *et al* (2006)
Specificity of Pap smear	96.8	96.3–97.3	Mayrand *et al* (2007)
Specificity of HPV DNA testing	87.5	85.0–89.9	Arbyn *et al* (2006)
Specificity of Pap smear and HPV DNA testing	87.3	87.3–90.4	Arbyn *et al* (2006)
			
*Utility value (6-month)*			
Normal population	1.00	1.00	Suárez *et al* (2008)
HPV	1.00	0.99–1.00	
CIN I	0.96	0.92–0.99	
CIN II/III	0.96	0.92–0.99	
Treated cancer	0.73	0.58–0.87	
Cured/follow-up cancer	0.94	0.62–0.97	
			
*Direct medical costs, NT$* b ,c			
* Screening test, NT$*			
Pap smear^d^	430.0		BNHI (2009)
HPV DNA testing	600.0		Market price
HPV DNA testing and Pap smear	1030.0		
			
*Diagnosis costs, NT$*			
Repeat Pap smear^e^	440.0		BNHI (2009)
Colposcopy (with biopsy)	2183.0		Debicki *et al* (2008)
			
*Treatment costs, NT$*			
CIN I	2347.0		Tang *et al* (2010)
CIN II/III	6956.0		
1st year of cervical cancer	232 389.0		
2nd year of cervical cancer	123 250.0		
3rd year of cervical cancer	167 009.0		
4th year of cervical cancer	95 645.0		
5th year of cervical cancer	113 627.0		

Abbreviations: CIN=cervical intraepithelial neoplasia; HPV=human papillomavirus; NHI=National Health Insurance.

a Sensitivity was defined as a screening positive result for patients with CIN I or worse in this model. Specificity was defined as a negative result for women in normal state.

b Range between −50% and +50% of the base-case value unless otherwise indicated.

c Direct medical costs were the costs associated with the use of health-care services covered by the NHI program.

d Pap smear screening procedures were (1) Pap smear sampling, (2) a pelvic examination, and (3) Pap smear cytological examination. Health-care providers are reimbursed NT$430 by the NHI system for these services.

e The procedures for a repeated Pap smear are (1) vaginal irrigation, (2) a pelvic examination, (3) obtaining a Pap smear sample, and (4) a Pap smear cytological examination. Health-care providers are reimbursed NT$440 by the NHI system for these services.

**Table 3:** Total life expectancy, total QALYs, total lifetime costs, and ICERs of all alternatives screening strategies in health care perspective

**Screening strategy^a^**	**Cancer cases, per 10^5^**	**Death of cancer, per 10^5^**	**Total undiscounted life expectancy**	**Total life expectancy^b^**	**Total undiscounted QALYs**	**Total QALYs^b^**	**Total undiscounted lifetime costs, NT$**	**Total lifetime costs, NT$^b^**	**ICER, NT$/QALYs^c^**	**ICER, NT$/ QALYs^d^**	**ICER, NT$/ QALYs^e^**
No screening	184.1	55.0	49.3586	25.5699	49.3339	25.5583	999	425	—	—	—
Pap, 5-y	118.2	34.8	49.3629	25.5713	49.3416	25.5612	4700	2352	659 966	659 966	Less costly but less effective
Pap, 3-y	102.9	30.2	49.3638	25.5716	49.3436	25.5620	6057	3045	708 189	888 718	Less costly but less effective
Pap, 1-y	87.6	25.6	49.3648	25.5719	49.3458	25.5629	8355	4242	837 171	Extended dominance	—
HPV-Pap, 5-y	70.0	20.7	49.3655	25.5721	49.3480	25.5636	11 084	5202	896 360	1 323 497	1 246 883
Combination, 5-y	65.2	19.3	49.3658	25.5723	49.3487	25.5639	12 457	6088	1 009 501	Extended dominance	1 757 905
HPV-Pap, 3-y	60.0	17.8	49.3661	25.5724	49.3499	25.5644	13 149	6261	955 254	1 357 692	1 302 645
Combination, 3-y	57.1	16.9	49.3663	25.5724	49.3501	25.5645	14 700	7301	1 103 724	Extended dominance	1 831 557
HPV-Pap, 1-y	54.6	16.3	49.3664	25.5725	49.3506	25.5647	15 377	7507	1 101 369	3 891 250	1 745 615
Combination, 1-y	53.2	15.9	49.3665	25.5725	49.3509	25.5648	17 031	8696	1 262 840	9 915 000	2 238 241

Abbreviations: ICER=incremental cost-effectiveness ratio; QALY=quality-adjusted life year.

a Pap refers to Pap smear as the primary screening test followed by colposcopy-guided biopsy for positive Pap smear results. HPV-Pap refers to HPV DNA testing as the primary screening test, using a Pap smear as a triage strategy for those who test positive for high-risk HPV types in HPV DNA testing. Combination refers to HPV DNA testing in combination with Pap smear as the primary screening test, using a Pap smear as a follow-up screening strategy for those who have positive Pap smear results or test positive for high-risk HPV types. 1-y, 3-y, and 5-y refer to three screening intervals of annually, every 3 years, and every 5 years for women 30–69 years of age, respectively.

b Life expectancy, QALYs, and costs are discounted at 3% per annum.

c Cost-effectiveness ratio calculated as the difference in cost divided by the difference in QALYs for each strategy compared with the no screening strategy.

d Cost-effectiveness ratio calculated as the difference in cost divided by the difference in QALYs for each strategy compared with next best strategy.

e Cost-effectiveness ratio calculated as the difference in cost divided by the difference in QALYs for each strategy compared with annual Pap smear alone strategy.
